# Micromastia with Abundant Lactation

**Published:** 1913-11

**Authors:** 


Micromastia With Abundant Lactation. Variot, *Jour. de Med. et de Chir. Prat.*, calls attention to the lack of relation between the size of the breasts and the functional activity. He cites a case of a woman of forty with well formed nipples but with grandular masses no larger than a silver dollar. She had nursed satisfactorily seven children.

